# Case-based evidence links increased SARS-CoV-2 diversity to impaired IFN-I response in severe COVID-19

**DOI:** 10.70962/jhi.20250019

**Published:** 2025-07-24

**Authors:** David Bussy, Kylian Trepat, Hadrien Regue, Valérie Cheynet, Laurence Generenaz, Louis Chauvelot, Jean-Christophe Richard, INTERFERICUS Study Group, Mehdi Mezidi, Hodane Yonis, Laurent Bitker, Guillaume Deniel, Ines Noirot, François Dhelft, Maxime Gaillet, Rosalie Schoux, Yorick Rodriguez, Florent Wallet, Donatien De Marignan, Auguste Dargent, Laurence Josset, Antonin Bal, Sophie Trouillet-Assant

**Affiliations:** 1 Hospices Civils de Lyon, Croix-Rousse Hospital, Medical Intensive Care Unit, Lyon, France; 2 Faculté de médecine Lyon-Est, Université de Lyon, Université Claude Bernard Lyon 1, Lyon, France; 3 Centre International de Recherche en Infectiologie (Team VirPath), Inserm U1111, Université Claude Bernard Lyon 1, CNRS UMR5308, ENS de Lyon, Lyon, France; 4 Joint Research Unit Hospices Civils de Lyon-bioMérieux, Hospices Civils de Lyon, Lyon Sud Hospital, Pierre-Bénite, France; 5 Laboratoire de Virologie, Institut des Agents Infectieux, Laboratoire associé au Centre National de Référence des virus des infections respiratoires, Hospices Civils de Lyon, Lyon, France; 6 GenEPII Sequencing Platform, Institut des Agents Infectieux, Hospices Civils de Lyon, Lyon, France; 7 Université Claude Bernard Lyon 1, INSA-Lyon, CNRS, INSERM, CREATIS UMR 5220, U1294, Villeurbanne, France

## Abstract

Severe COVID-19 with neutralizing anti–type I interferon autoantibodies correlates with high intra-host SARS-CoV-2 diversity. Findings suggest that impaired interferon-mediated viral control may facilitate the emergence of viral variants within individual patients during prolonged infection.

## Teaser

We present a case of increased within-host viral diversity linked to impaired type I interferon response during COVID-19.

## Introduction

Within-host viral diversity has been identified as a potential key factor driving the emergence of SARS-CoV-2 variants that drove successive waves of infection. This has been mostly described in highly immunocompromised patients with altered adaptive immunity, such as patients with cancer, under immunosuppressive treatment, or having received a transplantation. However, the adaptive immunity itself has not been associated with high within-host diversity in immunocompetent subjects (1). Instead, prolonged shedding of SARS-CoV-2 seems to be the major factor associated with high within-host diversity. The control of viral replication is driven by the type I interferon (IFN-I) response, a key player of innate immune response. Recent observations indicate that critically ill patients infected with SARS-CoV-2 frequently exhibit impairment of the IFN-I response (2). Consequently, we hypothesized that an altered IFN-I response could lead to uncontrolled viral replication and contribute to within-host viral diversity. The present study aimed to explore the association between innate immune response and the emergence of within-host SARS-CoV-2 genetic variants in a cohort of patients without known adaptative impairment, admitted to the Critical Care Unit (CCU) for acute respiratory failure.

## Results

### Study population

Forty SARS-CoV-2 nasopharyngeal samples (NPS) collected from 19 patients between November 2022 and January 2024 were sequenced in duplicate. Following the exclusion of 16 samples based on quality criteria (1 sample) or the absence of intra-host single nucleotide variants (iSNVs; 15 samples), iSNV was investigated in a total of 24 samples from 16 patients in the present study. NPS were collected from patients with a median (interquartile range [IQR]) age of 77 (69–84) years; 75% were male (12/16), and the mortality rate was 25% (4/16). Four patients were treated with remdesivir. The median [IQR] interval between the onset of symptoms and swab collection was 8 (4–9) days.

### Abundant iSNVs detected in one patient

**Figure 1.**
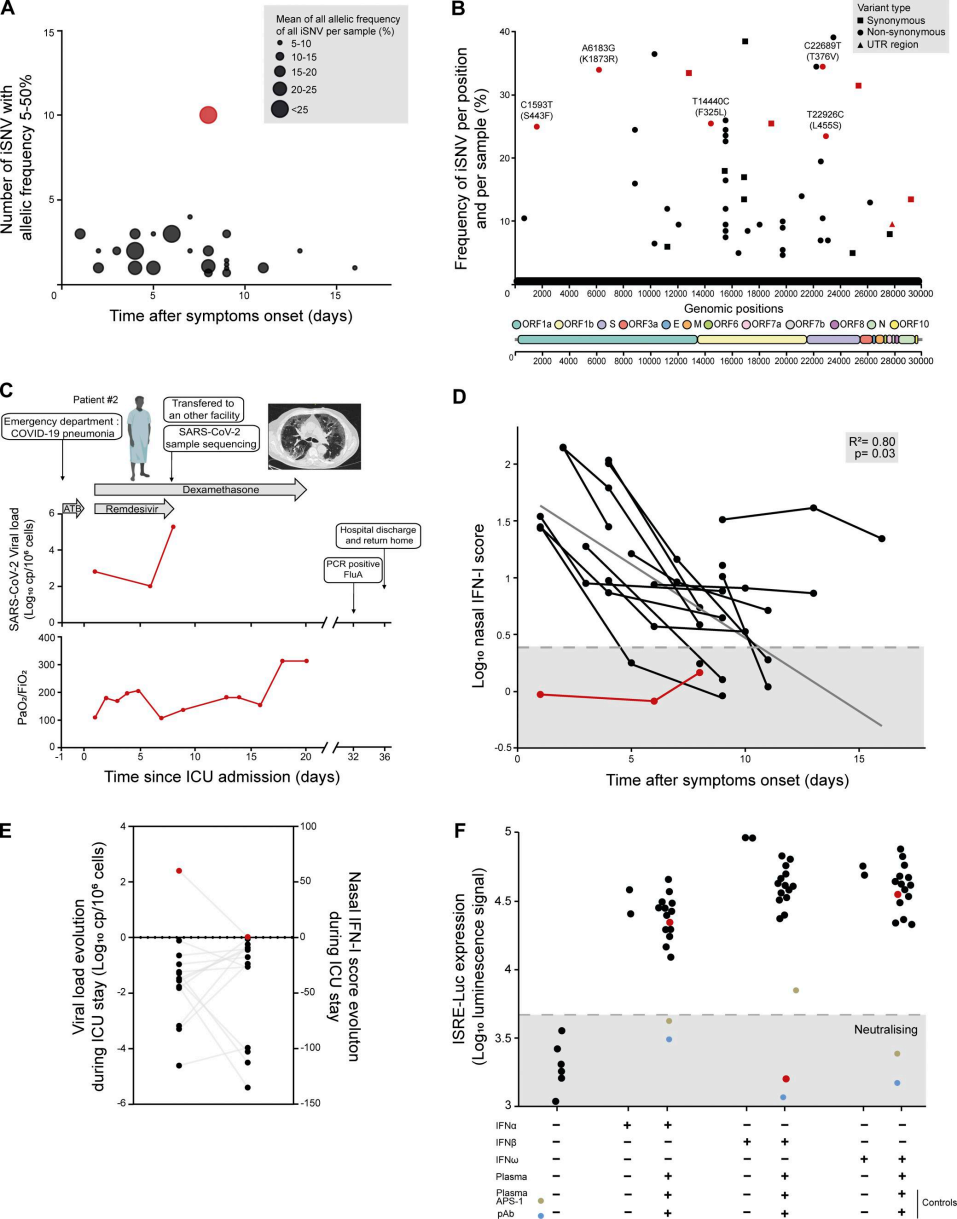
**Investigation of intra-host SARS-CoV-2 diversity in a cohort of patients admitted in CCU for severe COVID-19. (A)** Number of iSNVs and mean allelic frequency in each sample according to post-symptom delay. Red dot corresponds to the sample obtained from patient#2 sequenced at day 9 after CCU admission (8 days after remdesivir treatment). **(B)** Distribution of all iSNVs across the SARS-CoV-2 genome. Circles represent non-synonymous mutations, squares synonymous mutations, and triangles mutations in untranslated region (UTR). Non-synonymous mutations are showed with the Wuhan-Hu-1 genome as reference. **(C)** Medical history and evolution of SARS-CoV-2 viral load and PaO_2_/FiO_2_ ratio (red line) obtained from the NPS of the patient#2. A lower PaO_2_/FiO_2_ ratio indicates a more severe hypoxemic state. The patient had a medical history of diabetes, hypertension, dyslipidemia, as well as peripheral arterial occlusive disease and received four SARS-CoV-2 vaccination doses. Computed tomography (CT) scan was obtained 1 day prior to CCU admission and showed a “crazy paving” pattern with severe involvement (>50% of the lung parenchyma). A chest CT scan performed on day 10 after CCU admission revealed the appearance of a fibrotic-like pattern. Interestingly, patient#2 tested negative for SARS-CoV-2 on day 32 after CCU admission and simultaneously tested positive for influenza A virus (FluA) that can be associated with the alteration of immune antiviral defense caused by the presence of anti–IFN-β antibodies. He was discharged and returned home on day 36. The patient remained alive 90 days after inclusion. **(D)** Evolution of the nasal IFN-I score according to symptom onset during the CCU stay (*n* = 16 patients). A mixed-linear regression model was used with post-symptoms delay (days) as a fixed effect and individual slope and intercept as random effects. The grey line represents the predicted value of the fixed effect. The grey area represents the normal range defined in uninfected healthy volunteers. The P value indicates the statistical significance of the fixed effect. R-squared represents the conditional R-squared. **(E)** Association of viral load kinetics with nasal IFN-I score kinetics during CCU stay (*n* = 16). The evolution of viral load and nasal IFN-I score during CCU stay was assessed by calculating the differential between samples obtained at 7 days and 1 day after the inclusion (*n* = 10) or 3 and 1 days after the inclusion (*n* = 4). For 2 patients, only the sample from day 1 was available, and thus they are not represented in this figure. Grey lines connect the trajectories of both parameters for each patient. Red line/dots depicted values for patient with anti–IFN-β antibodies. **(F)** Representation of the luciferase activity under different IFN-I cytokines conditions, in the presence or absence of a patient’s plasma (each point corresponds to one patient). One plasma sample was missing for IFN-β and IFN-ω assay, and two plasma samples were missing for the IFN-α2 assay. The grey zone corresponds to neutralizing activity defined as the median plus 3 standard deviations of the control conditions luciferase activity (cells only), corresponding to a value of 3.67 log_10_ luciferase activity. Red dot corresponds to the sample obtained from patient#2. Patient suffering from autoimmune polyendocrinopathy candidiasis ectodermal dystrophy (APS-1) (positive control for anti–IFN-α and -ω autoantibodies, *n* = 1) and polyclonal antibody neutralizing all of IFN-α, -β, and -ω IFN (*n* = 1) (Tebubio, ref product 39000-1, Le Perray en Yvelines, France) were used as positive controls for IFN-I neutralization. The experiments were performed in duplicate, and the neutralizing activity of IFN-β was confirmed in an independent experiment. CCU, Critical Care Unit, ICU, Intensive Care Unit.

We identified 52 iSNVs, of which 40 were non-synonymous, resulting in a median [IQR] of 2 [1–3] iSNVs per sample at a median [IQR] allelic frequency of 13% [8–25%]. The sample obtained from patient#2 exhibited the highest number of iSNV, with 10 mutations at high allelic frequencies (9.5–34.5%; Fig. 1, A and B). Patient#2 had an infection with JQ.1 lineage according to pangolin classification. Interestingly, we identified in this sample the presence of two non-synonymous mutations in the spike gene, one of which is known to be a marker of JN.1 variant (L455S).

### Viral rebound despite high anti–receptor-binding domain (anti-RBD) IgG levels and antiviral treatment

The sample obtained from patient#2 was collected from a 79-year-old male admitted to the CCU for acute respiratory failure. The NPS containing high iSNV count was collected 1 wk after CCU admission. Prior samples could not be sequenced due to low viral load. Notably, this patient exhibited a viral rebound, reaching 5.2 log_10_ copies/10^6^ cells, despite high anti-RBD IgG levels at inclusion (12,576 binding antibody units per ml [BAU/ml]) and the administration of remdesivir throughout the CCU stay (Fig. 1 C). Interestingly, the PaO_2_/FiO_2_ ratio decreased during viral rebound. After CCU discharge, the patient was transferred to the Intensive Care Unit in a nonteaching hospital where he stayed for 11 days before then being transferred to a pneumology unit where a superinfection caused by influenza A was documented by PCR.

### Inadequate innate immune response due to IFN-β–neutralizing activity

We observed a consistently low IFN-I nasal score for patient#2 throughout the CCU stay, remaining below the positive threshold previously described in healthy volunteers (Fig. 1 D). The viral rebound was not associated with an increase in the IFN-I response, highlighting an inadequate innate immune response (Fig. 1 E). To explore a potential immune cause, we assessed plasma neutralization of IFN-I using a neutralization assay. Only plasma from patient#2 had strong neutralizing activity against IFN-β (10 ng/ml; Fig. 1 F).

## Discussion

To explore the relationship between an altered antiviral innate immune response and within-host viral variants, we conducted an analysis in a clinical cohort of SARS-CoV-2-infected patients admitted to the CCU with no prior history of adaptative immunosuppression. Considering all NPS included, we did not observe a high iSNV count (median of two mutation sites per sample). We identified one patient with the highest number of iSNVs, despite harboring an elevated anti-RBD IgG level. During his CCU stay, a viral load rebound associated with a decrease of PaO_2_/FiO_2_ ratio was observed without an associated increase of the IFN response. This patient’s viral load exceeded 4 logs 8 days after CCU admission, a level typically associated with an elevated IFN-I score in most critically ill COVID-19 patients (3). Plasma obtained from this patient had a high neutralizing capacity against IFN-β, indicative of the presence of IFN-β autoantibodies as previously identified in critical COVID-19 patients (2). This could explain the apparent absence of an IFN-I response. Of note, although anti–IFN-β autoantibodies are much less prevalent and less frequently associated with severe SARS-CoV-2 infections than anti–IFN-α and -ω autoantibodies (2), this case report highlights the role of those autoantibodies in the pathophysiology of SARS-CoV-2 infections.

Previous studies reported that SARS-CoV-2 infection in immunocompromised patients treated with remdesivir can lead to the emergence of viral variants harboring transmissible mutations conferring reduction in susceptibility to an RNA-dependent RNA polymerase (RdRP) inhibitor (https://covdb.stanford.edu/drms/rdrp/) (4). However, in both the consensus sequence and the iSNV, no mutation that is known to confer resistance to RdRP inhibitors was detected in patient#2, suggesting that the presence of anti–IFN-β autoantibodies, throughout uncontrolled replication, could also contribute to this variability. Moreover, vaccination has not been associated with an increase in the emergence of SARS-CoV-2 variants (1), suggesting that the patient’s vaccination status and the high level of anti-RBD level does not explain the high iSNV rate. Although heterotypic co-infection during the CCU stay could explain the high iSNV count, we are unable to test this hypothesis due to absence of eligibility criteria for prior sample obtained from patient#2.

In conclusion, host factors contributing to intra-host variant emergence are multiple, and we underlined that impairment of IFN-I response could be a significant contributor.

## Materials and methods

### Cohort

The INTERFERICUS cohort is a prospective multicenter study investigating the impact of IFN-I autoantibodies on viral load in critically ill patients. The trial was registered on https://ClinicalTrials.gov (NCT05536219), where inclusion and exclusion criteria are detailed. The study protocol was approved by a research ethics committee (Comité de Protection des Personnes Sud-Ouest et Outre-Mer II, 09/27/2022) under the IDCRB_2022-A01824-39, and opposition to use of personal data was sought from the patients or their representative.

### SARS-CoV-2 whole-genome sequencing

NPS collected from patients with acute respiratory failure induced by SARS-CoV-2 were selected for the present study. The SARS-CoV-2 load was determined from the NPS using SARS-CoV-2 R-gene kit (bioMérieux) as previously described (3). All NPS positive for SARS-CoV-2 with a cycle threshold ≤28 were sequenced in duplicate, using the Artic V4.1 NCOV-2019 Panel as previously described (5). Samples with ≥1,000,000 read count, ≥90% coverage of the consensus sequence, and mean depth at each position ≥500 were included in the present study. A threshold of 5% minor allele frequency for both replicates was used to identify iSNVs.

RNA from NPS was extracted in duplicate using the automated MGISP-960 workstation using MGI Easy Magnetic Beads Virus DNA/RNA Extraction Kit (MGI Tech) for the first extract and EMAG platform (bioMérieux) for the second extract. SARS-CoV-2 Artic V4.1 primers were tested on duplicate extracts from NPS at 10 µM final concentration. cDNA synthesis, amplification, and libraries were prepared using the COVIDSeq-Test (Illumina). Samples were sequenced with 100-bp paired-end reads using the NovaSeq 6000 Sequencing system SP flow cell.

Although contamination during sequencing could introduce artefacts, the quality controls (negative template) and the duplicate conditions performed minimize this possibility and cannot explain the elevated iSNV rate.

### FilmArray IFN panel

The IFN-I response was assessed using a FilmArray IFN-I pouch prototype, allowing the measurement of four IFN-stimulated genes and three housekeeping genes for signal normalization (this prototype has not been submitted to any regulatory agency for review at the time of writing). A total of 100 μl of diluted NPS was tested using the prototype IFN-I pouch, and the nasal IFN-I score was calculated as described previously (3).

### Neutralization assay

The blocking activity of anti–IFN-α2, -β and -ω AAbs was determined using a HEK293T reporter cell line constructed to react specifically to IFN-I by induction of luciferase synthesis. Cells were cultured in Dulbecco’s Modified Eagle Medium (Gibco) supplemented with fetal bovine serum at 10%. Sera were decomplemented (15 min/56°C). Subsequently, sera diluted 1/10 in duplicate were added to IFN-α2 (ref_130-093-874; Miltenyi), IFN-β (ref_300-02BC; PeproTech), or IFN-ω (ref_SRP3061; Sigma-Aldrich) at whether 10 or 1 ng/ml concentration to HEK293T cell (1.7 × 10^5^ cells/ml) in 96-well plate. After 24 h of incubation (37°C, 5% CO_2_), the firefly luciferase substrate (Bright-Glo, Promega) was added into the wells (100 μl), and luciferase activity was measured at least 5 min later on BioTek Synergy HTX (Agilent) using a 100-ms integration time.

### Measurement of anti-RBD IgG titers

Anti-RBD IgG titers were measured using the VIDAS SARS-CoV-2 IgG diagnostic kit (ref_423834; bioMérieux) and expressed in BAU/ml using the conversion factors provided by the manufacturer.

### Statistical analysis

Quantitative variables were expressed as median [IQR] and qualitative variables as number (percentage). Statistical analyses were performed using R version 4.3.2.

## Supplementary Material

Table S1 lists the INTERFERICUS Study Group members and affiliations.

## Data Availability

Requests for data should be made to the corresponding author. The datasets generated and analyzed during the current study are available from the corresponding author on reasonable request. The sequencing data are available under the BioProject accession number PRJNA1189494.
