# Supplementary material for: Case-based evidence links increased SARS-CoV-2 diversity to impaired IFN-I response in severe COVID-19
Source: J Hum Immun. 2025 Jul 24;1(3):e20250019. doi: 10.70962/jhi.20250019 (PMC13177429; doi:10.70962/jhi.20250019)
Supplement: Table S1 — lists the INTERFERICUS Study Group members and affiliations. [file jhi_20250019_tables1.pdf]

The INTERFERICUS Study Group: Mehdi Mezidi[1], Hodane Yonis[1], Laurent Bitker[1,2,3], Guillaume Deniel[1,2,3], Ines Noirot[1], François Dhelft[1], Maxime Gaillet[1], Rosalie Schoux[1], Yorick Rodriguez[1,2], Florent Wallet[4], Donatien De Marignan[4], Auguste Dargent[4], Laurence Josset[5].

1 Hospices Civils de Lyon, Croix-Rousse Hospital, Medical Intensive Care Unit, Lyon, France

2 Université de Lyon, Université Claude Bernard Lyon 1, Faculté de médecine Lyon-Est, Lyon, France.

3 Univ Lyon, Université Claude Bernard Lyon 1, INSA-Lyon, CNRS, INSERM, CREATIS UMR 5220, U1294, Villeurbanne, France

4 Service d'Anesthésie Médecine Intensive-Réanimation, Hospices Civils de Lyon, Hôpital Lyon Sud, 165 Chemin du Grand Revoyet, Pierre-Bénite, Lyon, France.

5 CNR des Virus des Infections Respiratoires, Institut des Agents Infectieux, Hospices Civils de Lyon, Lyon, France; Virpath, Centre International de Recherche en Infectiologie, Université de Lyon, Inserm U1111, CNRS UMR5308, École Normale Supérieure de Lyon, UCBL, Lyon, France
